# The potential of oxygen and nitrogen species-regulating drug delivery systems in medicine

**DOI:** 10.3389/fbioe.2022.973080

**Published:** 2022-08-30

**Authors:** Michał Sołtan, Dorota Bartusik-Aebisher, David Aebisher

**Affiliations:** ^1^ English Division Science Club, Medical College of The University of Rzeszów, Rzeszów, Poland; ^2^ Department of Biochemistry and General Chemistry, Medical College of The University of Rzeszów, Rzeszów, Poland; ^3^ Department of Photomedicine and Physical Chemistry, Medical College of The University of Rzeszów, Rzeszów, Poland

**Keywords:** oxygen species, nitrogen species, biomaterials, biomedical applications, oxygen

## Abstract

The focus of this review is to present most significant advances in biomaterials used for control of reactive oxygen/nitrogen species (ROS/RNS, RONS) in medicine. A summary of the main pathways of ROS production and the main pathways of RNS production are shown herein. Although the physiological and pathological roles of RONS have been known for at least 2decades, the potential of their control in management of disease went unappreciated. Recently, advances in the field of biochemical engineering and materials science have allowed for development of RONS-responsive biomaterials for biomedical applications, which aim to control and change levels of reactive species in tissue microenvironments. These materials utilize polymers, inorganic nanoparticles (NPs), or organic-inorganic hybrids. Thus, biomaterials like hydrogels have been developed to promote tissue regeneration by actively scavenging and reducing RONS levels. Their promising utility comes from thermo- and RONS-sensitivity, stability as a delivery-medium, ease for incorporation into other materials and facility for injection. Their particular attractiveness is attributed to drug release realized in targeted tissues and cells with elevated RONS levels, which leads to enhanced treatment outcomes and reduced adverse effects. The mechanism of their action depends on the functional groups employed and their response to oxidation, and may be based on solubility changes or cleavage of chemical bonds. When talking about antioxidants, one should also mention oxidative stress, which we call the imbalance between antioxidants and reactive oxygen species, which occurs due to a deficiency of endogenous antioxidants and a low supply of exogenous antioxidants. This study is a review of articles in English from the databases PubMed and Web of Science retrieved by applying the search terms “Oxygen Species, Nitrogen Species and biomaterials” from 1996 to 2021.

## Introduction

Reactive species, also commonly called free radicals, is a term that describes atoms and functional groups with one or more unpaired electrons. Due to this odd number of electrons, free radicals are potent electrophiles, making them unstable and highly reactive. The dire atomic need for obtaining an even number of electrons on the valence shell and the resultant stability, drives these reactive species to react with (“attack”) other molecules to attain the needed electrons. In turn, this process leads to the attacked molecule losing electrons and it becoming a free radical itself. The aforementioned electron-capturing process continues, forming a cascade of reactions that usually leads to a degenerative process, mostly cell death. Along the way, molecules like lipids, proteins and nucleic acids may fall victims to reactive species (Mittal et al., 2014). The sources of free radicals in the human organism can be exogenous (e.g., UV radiation, alcohol, pesticides, heavy metals, some drugs) or endogenous (usually from oxidative processes in mitochondria, peroxisomes, and the endoplasmic reticulum) (Yoboue et al., 2018; Burtenshaw et al., 2019). To counteract free radicals, cells employ many enzymes and other non-enzymes that scavenge these highly reactive molecules and neutralize them; all these enzymes and non-enzymes are collectively referred to as the antioxidant defense system. Under normal conditions, there is a balance between oxidants and antioxidants, meaning the free radicals are neutralized nearly immediately and at a sufficient rate. However, an imbalance between these two components can occur when there is a greater production of free radicals and the antioxidant capacity is exceeded, or when the antioxidant defense system itself is impaired (Birben et al., 2012). This in turn leads to the state of oxidative stress that commonly results in overall aging, but may also result in many diseases (rheumatoid osteoarthritis, atherosclerosis, asthma, cataract, Alzheimer’s disease, Parkinson’s disease, multiple sclerosis and many cancers) (Phaniendra et al., 2015). Due to oxygen and nitrogen being among the primary building elements of human organisms, and because of the many redox reactions in which these two elements are involved, this article will focus on their reactive forms - reactive oxygen/nitrogen species (RONS).

RNS/RNO sensitive biomaterials can be used as a RONS regulating drug delivery systems due to several advantages resulting from their structure. The advantages that make RNS/RNO sensitive biomaterials attractive include biodegradability, biocompatibility, improved circulation and reduced toxicity (Joshi-Barr et al., 2014). Biomaterials often elicit a strong inflammatory response *in vivo* due to an immune response to their presence which is likely to cause higher levels of oxidative stress. Oxidative stress using drug-containing nanoparticles is yet to be explored. Oxidative stress can be managed during the implantation of biomaterials to promote their integration. Today, the most common approach to dealing with oxidative stress is to use antioxidants. Biomaterials such as collagen and elastin are natural polymers and exhibit improved biological properties such as adhesion and proliferation compared to *in vitro* synthetic materials. Factors influencing this response include manufacturing processes, rate of material degradation, and the presence of antigens (Troy et al., 2021). Extracellular matrix (ECM) degradation may also induce the formation of oxidants indirectly as ECM fragments have been shown to promote immune cell recruitment. However, some natural polymers used in scaffolds can break down into products that act as antioxidants. ECM scaffolds are typically processed by decellularization and chemical cross-linking methods to remove or mask antigenic epitopes, DNA, and Damage Associated Molecular Pattern (DAMP). Inadequate removal of DAMP from biological materials can lead to oxidative stress when released *in vivo* (Aamodt and Grainger, 2016). Moreover, for example, glycosaminoglycans (such as chondroitin sulfate and hyaluronic acid) have been identified as antioxidants capable of reducing free radicals, protecting both cells and materials from ROS damage (Shafiq et al., 2021).

## Search strategy and select criteria

A search focused on the Oxygen Species, Nitrogen Species and biomaterials was done on Pub-med, and Scopus from inception (1973) to July 2022. The study was performed based on the Preferred Reporting Items for Systematic Reviews and Meta-Analyses (PRISMA) guidelines (Page et al., 2021). The search term included was as follows: “Oxygen Species, Nitrogen Species and biomaterials”. Three authors undertook the task of identification data. Any discrepancies be-tween the reviewers were resolved by the third author. The authors of the reviews worked on the basis of an agreed scheme, distinguishing: title, language of the work, abstract and access to the entire article. Duplicate records were removed first.

**FIGURE 1:**
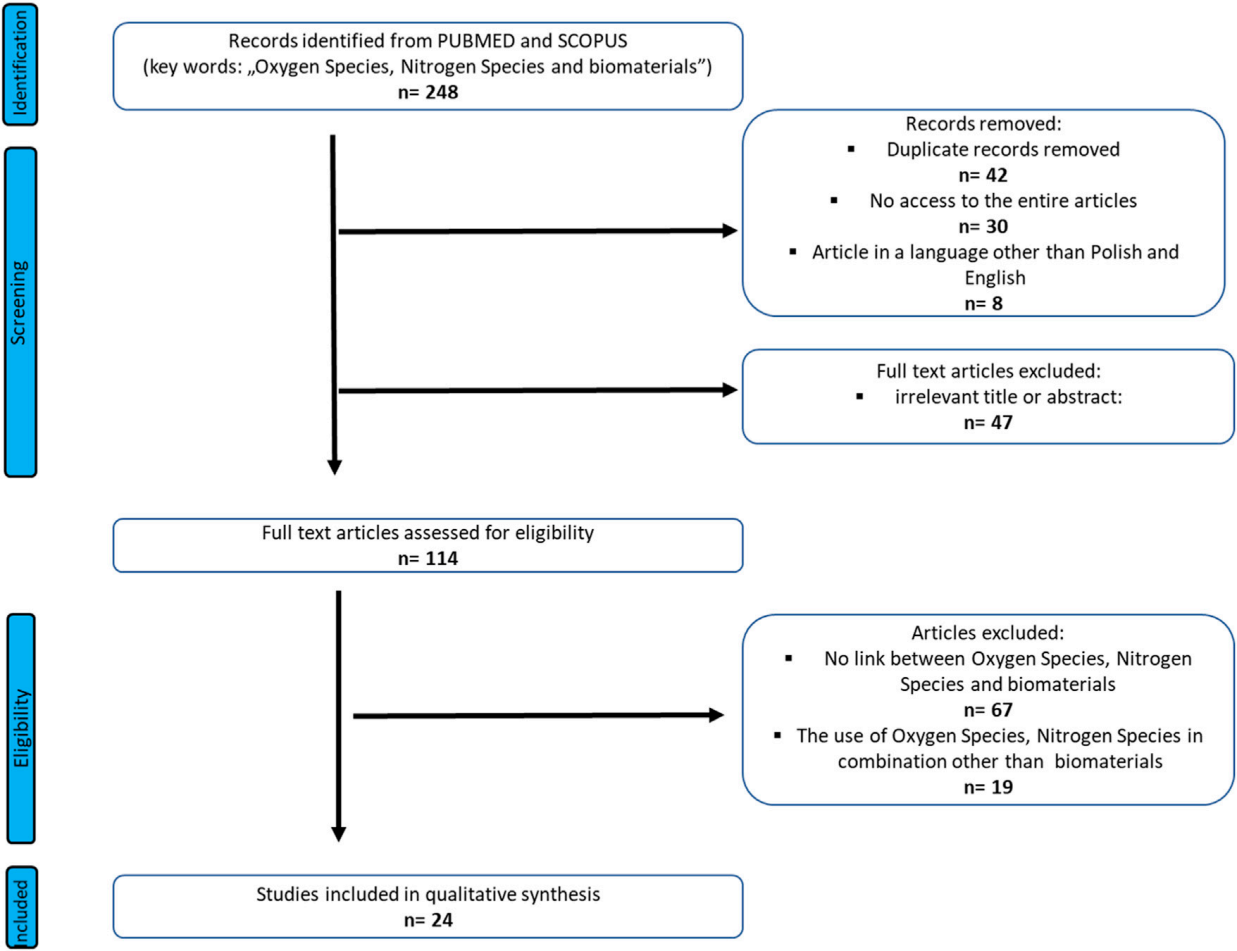
PRISMA flow diagram of included studies.

Full-text and accessible articles were further analyzed. In order to minimize the selection bias, the inclusion and exclusion criteria were established as follows (Figure 1):

### Inclusion criteria

Oxygen Species and biomaterials;
Nitrogen Species and biomaterials;
·Oxygen Species, Nitrogen Species and biomaterials Regulating Drug Delivery Systems in Medicine, have been included in the review;
·both Oxygen Species, Nitrogen Species and biomaterials were included in the review

### Exclusion criteria

·no analysis of the relationship between Oxygen Species, Nitrogen Species and biomaterials
·no analysis of the relationship between Oxygen Species, Nitrogen Species and biomaterials

## Reactive oxygen species

Reactive oxygen species (ROS) are oxygen-containing molecules with one or more unpaired electrons. Due to human organisms respiring aerobically, most of these free radicals are formed in mitochondria, as molecular oxygen is reduced during electron transport in the electron transport chain (ETC). The central ROS is superoxide anion (O_2_^−•^); although not strongly reactive, it is a precursor for the formation of other, stronger reactive forms and may propagate other oxidative chain reactions. Due to such dangerous potential, superoxide dismutase (SOD) immediately converts it into hydrogen peroxide (H_2_O_2_), a less toxic non-radical (Turrens, 2003). It still may reverse back to superoxide anion and in combination with the fact that hydrogen peroxide is an uncharged molecule and may leak from mitochondria into cytoplasm, it is also crucial to rapidly neutralize it. Thus, enzymes catalase (CAT) and glutathione peroxidase (GSH-Px) further dismutate hydrogen peroxide into water (H_2_O) (Bienert et al., 2006). Along the way, superoxide anion may reduce some transition metals, particularly Fe^3+^ into Fe^2+^, which in turn may react with hydrogen peroxide to form hydroxyl radical (OH^•^) (Vidrio et al., 2008). Hydroxyl radical is amongst the most reactive and deleterious free radicals, as it causes DNA/RNA damage, protein modification and lipid peroxidation, thus being the main contributor to oxidative stress (Scarcello et al., 2020). Aside from mitochondria, peroxisomes are also associated with substantial ROS production and neutralization, due to the presence of many oxygenases, superoxide dismutases, and catalase (Sandalio et al., 2013). Despite such noxious nature, stable and controlled nanomolecular concentrations of ROS have certain physiologic functions. Although it is not clear to what extent they are useful under normal cellular conditions, cellular stress induces ROS production and their utilization as signaling transductors (Schieber and Chandel, 2014). The main physiological functions of ROS include: 1) direct antimicrobial activity against pathogens in macrophages and neutrophils, 2) cytokine-activated signaling in many pro-inflammatory pathways, primarily through induction of MAPK, STAT1, STAT6 and NFκB, 3) post-translational regulation of many proteins and enzymes through thiol group oxidation, 4) p53-controlled apoptosis, 5) transcriptional adaptation to hypoxia, through stabilization of HIFs (Johnson et al., 1996; Movafagh et al., 2015; Rendra et al., 2019; Finelli, 2020; Herb and Schramm, 2021). ROS responsive polymers are ideal candidates for the development of stimuli-responsive biomaterials for target therapies. Among different ROS-responsive polymers, those containing thioether groups are widely investigated in the biomedical field due to their hydrophobic to hydrophilic phase transition under oxidative conditions (Criado-Gonzalez and Mecerreyes, 2022). ROS play important roles in cell signaling pathways, while increased production of ROS may disrupt cellular homeostasis, giving rise to oxidative stress and a series of diseases. Utilizing these cell-generated species as triggers for selective tuning polymer structures and properties represents a promising methodology for disease diagnosis and treatment (Xu et al., 2016). ROS-responsive polymer carriers allow the targeted delivery of drugs, reduce toxicity and side effects on normal cells, and control the release of drugs, which are all advantages compared with traditional small-molecule chemotherapy agents (Gao and Xiong, 2021). Polyoxalate (POx) and copolyoxalate (CPOx) smart polymers are topics of interest the field of inflammation. This is due to their drug delivery ability and their potential to target reactive ROS and to accommodate small molecules such as curcumin, vanilline, and p-Hydroxybenzyl alcohol (Yao et al., 2019). Recently, ROS-responsive biomaterials have been identified as a type of promising therapeutic substance to alleviate oxidative stress in tissue microenvironments, and for use as a vehicle triggered by inflammatory diseases to realize drug release under physiological oxidative microenvironments. The various applications of ROS-responsive biomaterials in tissue regeneration and disease therapy, such as cardiovascular diseases, osteoarthritis, chronic diabetic wounds, inflammatory bowel disease and other inflammatory diseases, are summarized (Swami Vetha et al., 2021).

## Reactive nitrogen species

Reactive nitrogen species (RNS) are all derived from nitric oxide (•NO) reacting with superoxide anion (O_2_^•−^) (Ronzio, 2020). Nitric oxide is a free radical itself and is produced through enzymatic activity of three isoforms of NO synthase (NOS), namely endothelial NOS (eNOS), neuronal NOS (nNOS), and inducible NOS (iNOS) (Costa et al., 2016). Although not a potent radical, nitric oxide may combine with superoxide anion to produce peroxynitrite (ONOO^−^), which aside to hydroxyl radical, is regarded as the second most reactive radical. Due to its powerful oxidizing and nitrating character, peroxynitrite may attack proteins and DNA/RNA, causing alterations to their functions and subsequent cellular damage (Islam et al., 2015). Additionally, nitric oxide may combine with molecular oxygen to form dinitrogen trioxide (N_2_O_3_), which in turn may nitrosate thiols of amino acids, also leading to loss of protein functionality (Jourd’heuil et al., 2003). Similarly, to ROS, RNS also display certain physiological functions when kept within safe concentrations. These functions include: 1) vasodilation produced by nitric oxide through activation of soluble guanylyl cyclase (sGC) and second messenger-mediated decrease of Ca^2+^ release, 2) direct antimicrobial activity against pathogens in macrophages and neutrophils, 3) signaling in many pro-inflammatory pathways, primarily through activation of TLR4 and NF-κB, 4) post-translational regulation of many proteins and enzymes, through thiol group oxidation, nitration and nitrosation, 5) apoptosis by inducing NF-κB, BAX and BAK, as well as activating caspases-3 and -9, 6) additionally, nitric oxide prevents platelet aggregation and leukocyte adhesion to endothelial walls (Ryan et al., 2004; Snyder et al., 2009; Rathnasamy et al., 2014; Gao et al., 2018; Kollau et al., 2018; Fang and Vázquez-Torres, 2019). These materials utilize polymers, nanoparticles (NPs), or gel systems.

## Reactive oxygen species-sensitive drug delivery systems

Since ROS and RNS are usually overproduced in inflamed tissues and tumors, researchers have been inspired to harness this potential for designing site- and stimuli-specific drug delivery systems (DDSs). Thus, two main types of DDSs can be distinguished: (1) DDSs with ROS-mediated solubility switch, and (2) DDSs with ROS-mediated degradation (Tao and He, 2018). Reactive oxygen species-mediated solubility switch DDSs (1) base their mode of action on the change in the solubility of the polymer in oxidative microenvironments. Upon oxidation of functional groups (amphiphilic, typically containing thioether, selenide, or tellurium), side chains transition from hydrophobic to hydrophilic, and the carried drug is released (Gao and Xiong, 2021). The main solubility switch DDSs are presented in Table 1.

**TABLE 1:** Main ROS-responsive solubility switch DDSs (Yao et al., 2019).

ROS-responsive linker	Chemical structure and oxidation	Material format	Ref
Poly(propylene sulfide)	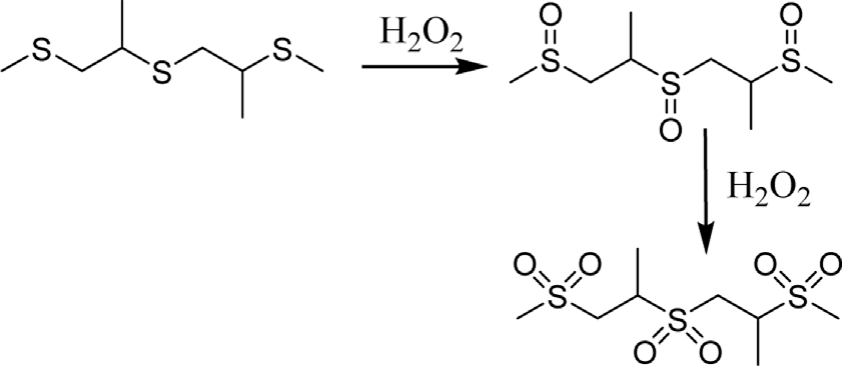	Polymeric micelles	Gupta et al. (2012)
Thioether	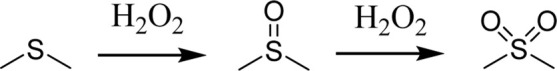	Polymeric NPs	Gong et al. (2019)
Selenium-containing polymers	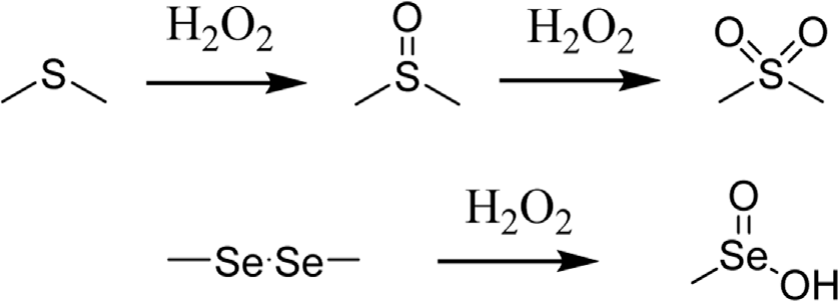	Polymeric aggregates	Ma et al. (2010)
		Polymeric micelles	Ji et al. (2013)
Tellurium-containing polymers	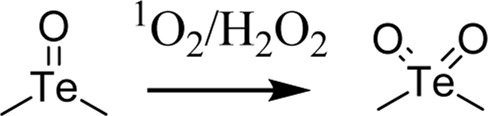	Spherical aggregates	Wang et al. (2015)

The first DDSs of this type was developed by Hubbell *et al.* in 2004. By means of anionic ring opening polymerization, a triblock ABA copolymer was prepared, with hydrophilic polyethylene glycol (PEG) as A block and hydrophobic poly(propylene sulfide) PPS as B block. This PEG-b-PPS copolymer was found to self-assemble into stable U-shaped vesicles in aqueous solution through hydrophilic/hydrophobic interactions. Upon treatment with 10% H_2_O_2_, hydrophobic sulfide is oxidized to more hydrophilic sulfoxide or sulfone, leading to destabilization of the vesicle and release of the carried agent (Napoli et al., 2004). However, PPS is not responsive to superoxide and thus Tirelli *et al.* have conjugated SOD with PEG-b-PPS copolymer. That way, PPS-PEG-SOD micelles can scavenge and react with both superoxide and hydrogen peroxide (Hu and Tirelli, 2012). For direct measure of applicability in drug delivery, Cheng *et al.* designed free-blockage mesoporous silica nanocarriers with hydrophobic phenyl sulfide groups (MSNs-PhS). As the phenyl sulfide groups oxidized to phenyl sulfoxide or phenyl sulfone, wettability of the nanopores increased and doxorubicin (DOX) was gradually released. This study revealed great potential of MSNs-PhS for selective chemotherapy, as DOX was released human breast adenocarcinoma (MCF-7) cells with high levels of ROS, and not in in normal human umbilical vein endothelial cells (HUVECs) with low ROS levels (Cheng et al., 2017). In 2020, Ford et al. designed a diblock copolymer for localized release of loaded diflunisal, as therapeutic approach to combating osteomyelitis caused by *Staphylococcus aureus*. Through RAFT polymerization the PPS_135_-*b*-p(Cy7_1_-*ran*-DMA_149_) nanoparticles were produced, where DMA unit composed the hydrophilic corona and the PPS unit constituted hydrophobic ROS-responsive core in close association with loaded drug. Upon parenteral administration of nanoparticles with diflunisal into mice subjected to osteomyelitis, polymer preferential distributed to infected femurs. There, owning to the inflamed milieu, PPS moiety underwent ROS-mediated oxidation, which resulted in change of core character from hydrophobic to hydrophilic and final release of diflunisal (Ford et al., 2021).

ROS-mediated degradation DDSs (2) base their mode of action on polymer chain breakdown induced by oxidative species and subsequent drug release. Functional groups serving as linkers are characterized by selective oxidation by ROS and rapid cleavage. Importantly, the generated byproducts are easily metabolized (Wilson et al., 2010). The main degradable DDSs are presented in Table 2.

**TABLE 2:** Main ROS-induced degradation DDSs (Yao et al., 2019).

ROS-responsive linker	Chemical structure and oxidation	Material format	Ref
Poly(thioketal)	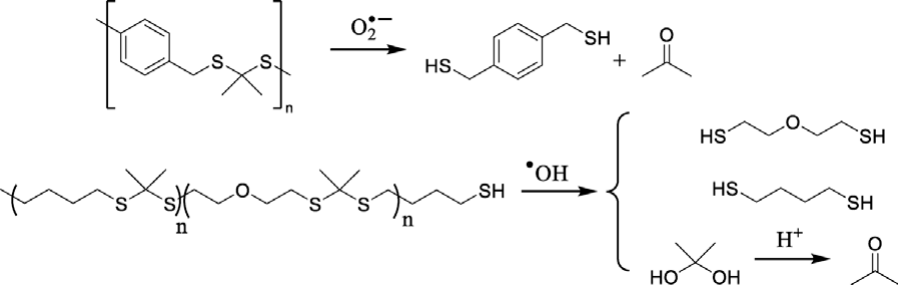	Polymeric NPs	Wilson et al. (2010)
		Polymeric scaffolds	Martin et al. (2014)
Arylboronic ester- containing polymers	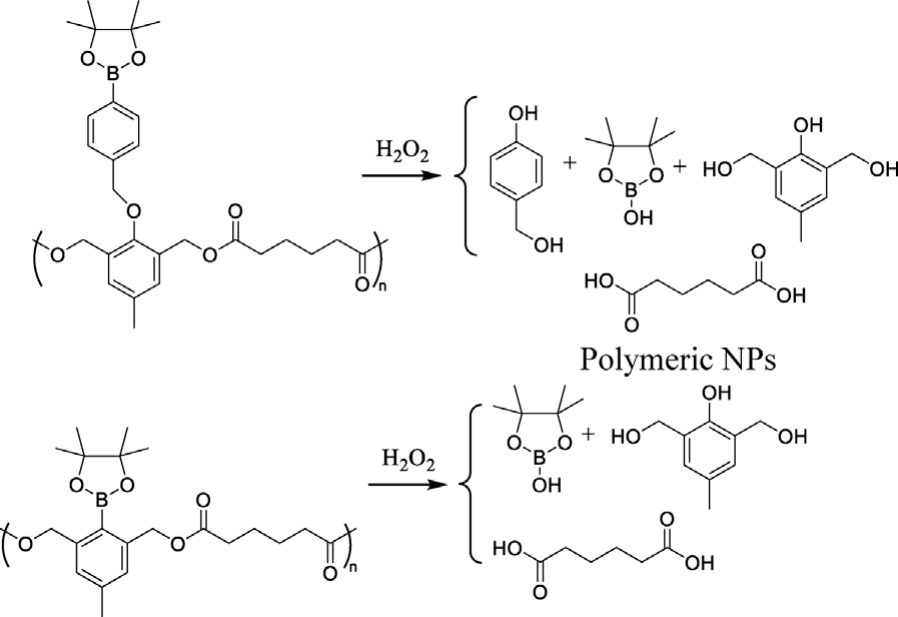	Polymeric NPs	de Gracia Lux et al. (2012)
		Polymeric NPs	
Poly(proline)	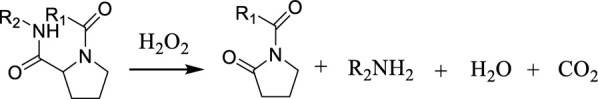	Polymeric scaffolds	Yu et al. (2011)

The most notable DDSs of this kind are polymers utilizing thioketal groups as linkers, since they show high sensitivity to different ROS species, including H_2_O_2_, •OH, and O_2_^−•^ (Shim and Xia, 2013). In 2010 Wilson et al. developed poly(1,4-phenylene-acetone dimethylene thioketal) (PPADT) through step-growth polymerization, intended for treatment of gastrointestinal inflammatory diseases. Loaded with tumor necrosis factor-alpha (TNFα)-siRNA, these nanoparticles were administered orally and showed stability in acidic and basic environments, as well as resistance to protease-catalyzed degradation. Those properties in combination with sensitivity to ROS allowed for relatively precise delivery of carried agent into the inflamed intestinal tissue (Wilson et al., 2010). As determined by Pu et al. in a different study, a similar PPADT polymer used for anti-inflammatory therapy responded to H_2_O_2_ in concentration as low as 1 mM, releasing 50% of the drug within 4 h (Pu et al., 2014).

## Discussion

Overproduction of RONS in tissue microenvironments is the main factor impeding normal healing and regeneration. Inflammatory responses induce DNA/protein damage and cell apoptosis, as well as hinder endogenous stem cells and macrophages.

Zhao et al. (2020) designed a hydrogel with ability to scavenge ROS in difficult-to-heal diabetic wounds with coexisting bacterial infections that further impede regeneration. First, the ROS-responsive linker, N1-(4-boronobenzyl)-N3-(4-boronophenyl)-N1,N1,N3, N3-tetramethylpropane-1,3-diaminium (TPA), was synthesized by quaternization reaction and then mixed with poly(vinyl alcohol) (PVA). The created PVA-TPA hydrogel showed ROS-scavenging ability; testing, where the hydrogel was incubated with H_2_O_2_ and titanyl sulfate as a H_2_O_2_-responsive probe, showed elimination of 65% of H_2_O_2_ within 1 h and nearly 100% within 24 h. Similar results were demonstrated *in vitro* testing, where the largest reduction of intracellular ROS levels was observed in hydrogel group combined with human umbilical vein endothelial cells (HUVECs) previously incubated with H_2_O_2_. For direct measure of healing properties, *in vivo* testing was conducted in mouse animal models. Inflammation was induced by injecting lipopolysaccharide (LPS), a cell wall component of Gram-negative bacteria, into mouse back skin. Photoacoustic (PA) imaging with the H_2_O_2_-specific Lipo@HRP&ABTS nanoprobe, showed that hydrogel could continuously decrease the ROS levels triggered by LPS. For assessment of wound closure-promoting abilities, dorsal wounds were created in mouse back skin, which were later treated with PVA-TPA hydrogel, and TPA and PVA separately. At day 8, 60% wound closure was noted in wounds treated with hydrogel, as opposed to closure of only 28% and 33% in TPA and PVA groups respectively. Additionally, aiding to promotion of wound healing, PVA-TPA hydrogel was found to reduce pro-inflammatory cytokines (interleukin-6 (IL-6), tumor necrosis factor (TNF-α), interleukin-1β (IL-1β), interleukin-23 (IL-23) and monocyte chemotactic protein 1(MCP-1)), increase the percent of M2 phenotype macrophages, and stimulate angiogenesis and collagen synthesis around the wound.

In an even more complex and effective approach to tissue repair, stem cells are delivered to the site of damage. Particularly, undifferentiated mesenchymal stem cells (MSCs) exhibit high potential in cell therapies, as they possess immunomodulatory functions through the secretion of paracrine factors that can positively direct tissue regeneration. Unfortunately, the main limiting factor in utility of this type of therapy is the poor survival of delivered cells due to the host’s immune response and production of high levels of RONS. Thus, Dollinger et al. developed an ABC triblock polymeric hydrogel, with poly(propylene sulfide)(PPS) as block A, N,N-dimethyl acrylamide (PDMA) as block B, and N-isopropylacrylamide (PNIPAAM) as block C (PPS-block-PDMA-block-PNIPAAM, PDN). Forming the core of the micelle, PPS enabled drug loading and its controlled ROS-mediated release. PDMA stabilized the hydrophilic side-chains and prevented syneresis of the assembled gels, while PNIPAAM provided for thermal gelation properties. Moreover, because delivered cells often suffer from poor adhesion due to lack of reinforcing structures, type 1 collagen (T1C) was added to provide for cellular adhesion motifs. *In vitro* testing on NIH 3T3 mouse fibroblasts grown in tissue culture plates, showed that PDN hydrogels scavenge H_2_O_2_. Additionally, fibroblasts suspended within PDN hydrogel + collagen composite were injected onto a heated surface and remained exclusively associated with the gelled hydrogel and viable for at least 24 h. To assess protective abilities against ROS-induced toxicity, hMSCs were encapsulated in PDN hydrogel, hydrogel without PPS (thus, no ROS-scavenging ability) and collagen gel, all of which were then cultured for 24 h and then treated with H_2_O_2_. In the PDN group, no significant change in the number of viable cells was noted, as opposed to considerable decrease in two other groups, and the viability was maintained at 80% or higher for at least 6 days in the presence of cytotoxic H_2_O_2_ concentrations. Such results confirm that PDN hydrogel possesses cyto-protective properties against ROS-induced apoptosis and may be a useful agent in increasing the feasibility of cell therapies (Dollinger et al., 2017). More recently, Yi et al. (2021) synthesized a modified liposome for localized NIR irradiation-triggered DOX release. In this polymer, boronic acid was used as ROS-sensitive moiety and conjugated with DOX and indocyanine green (ICG). Then encapsulated into PEG-modified liposomes, yielding Lipo/pB-DOX/ICG. *In vivo* testing where Lipo/pB-DOX/ICG was injected into mice with MDA-MB-231 tumors showed accumulated of liposomes at the tumor site within 24 h. Under 808 nm laser irradiation, ICG generated ROS which selectively cleaved boronate prodrug, releasing DOX.

### Reactive nitrogen species sensitive drug delivery systems

Similarly to ROS-DDS, polymeric systems have been designed, where the mechanism of loaded drug release was driven by NO- or ONOO^−^ -mediated oxidation of RNS-sensitive moiety and subsequent solubility change or degradation. Table 3. Shows main RNS-DDS.

**TABLE 3:** Main RNS-responsive DDSs (Zhao et al., 2021).

RNS-responsive linker	Chemical structure and oxidation	Material format	Ref
o-phenylenediamine	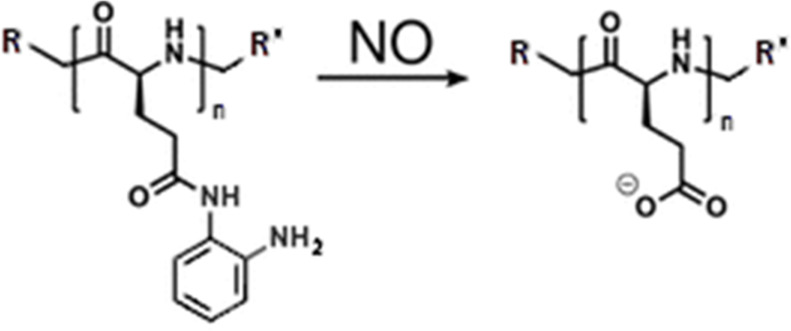	Liposomes	Wang et al. (2019)
		Nanofilaments	Liu et al. (2020)
		Micelles	Lu et al. (2020)
0 trifluoromethyl ketone	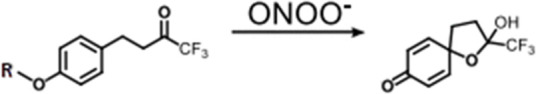	Polymersomes	Zhang et al. (2016)

In the past 2 decades, o-phenylenediamine has been evidenced as a highly selective and efficient reductor group of NO in the presence of O_2_, owning to its electron rich character (Plater et al., 2001). Since then, many fluorescent probes containing this moiety have been in common for *in vitro* and *in vivo* NO detection. A significant development was done in 2014 by Hu et al. who designed a thermoresponsive copolymers which exhibited considerable solubility change, with subsequent self-assembly into spherical micelles and fluorescence upon exposure to mild levels of NO (0–48 μM). Such self-assembly was attributed to NO-triggered formation of amide-substituted benzotriazole intermediates and urea-functionalized benzotriazole derivatives, with concomitant change in lower critical solution temperature (LCST). Later in 2019, Wang et al. produced a liposome with o-phenylenediamine-containing lipid embedded into its phospholipid bilayer, which further encapsulated L-arginine (l-Arg)/DOX-loaded gold-copper sulfide yolk–shell nanoparticles (_AD_Au@CuS YSNPs) to form _ADL_Au@CuS YSNPs (Wang et al., 2019). Using 808 nm NIR laser irradiation, ROS generation was induced, which then drove conversion of L-arginine into NO. Timed release of DOX was achieved by progressive regional destabilization of phospholipid bilayer by NO and molecular scaffold limit, allowing for final DOX release when liposome structure was destabilized severely enough. *In vivo* testing on mice elucidated this programmable liposome as a viable target therapy of DOX-resistant MCF-7/ADR cells and possible treatment option for other multidrug resistance (MDR) cancers. Next, Liu et al. exploited the nature of o-phenylenediamine by developing a block polymer poly(ethylene oxide)-b-poly(o-phenylenediamine L-glutamate) (PEO-b-PEOPA) through ring-opening polymerization (Liu et al., 2020). The obtained polypeptide chains exhibited self-assembly behavior, forming nanofilaments, which could then be cleaved by a biologically relevant level of NO into comparably small disassemblies within 3 h. Measurement of nanocarrier capacity and load release showed most of PEO-b-PEOPA efficiently releasing Rhodamine 6G under 40 μM (2.0 equiv.) of NO stimulus within 30–90 min. Additionally, Lu et al. prepared a system for targeted antibiotic delivery, by conjugating levofloxacin (LF) and hyaluronic acid (HA) via NO-sensitive o-phenylenediamine as linker, yielding HA-NO-LF nanomicelles (Lu et al., 2020). *In vitro* drug release testing demonstrated total drug release efficiency of nearly 70% achieved within 6–15 h in the presence of NO. Evidenced to easily enter macrophages via CD44 mediated endocytosis, therapeutic efficacy of these micelles was then tested *in vivo* in pneumonia-induced mice infected with *Staphylococcus aureus,* which showed greatest bactericidal effect in the group with HA-NO-LF and NO-stimulus present. Growing research into NO as an endogenous biosignal for RNS-DDSs has also brought focus onto ONOO^−^ as a possible exploitable trigger and prompted search for a ONOO^—^selective linker group. However, number of studies reporting development of ONOO^—^-specific nanoparticles seems to be currently limited. So far, the only notable invention in this category has been done by Zhang et al. (2016) in 2016, who utilized ONOO^—^-selective and specific cleavage of trifluoromethyl ketone (TFK) moiety and developed a block polymer poly(ethylene oxide)-b-PMATFK (PEO-b-PMATFK). In aqueous solution, this copolymer exhibited self-assembly into vesicular polymersomes, which then disassembled when subjected to ONOO^—^ (35 μM, 5 equiv). Additionally, this disassembly ability was dose-dependent, allowing for regulation of the vesicle dissociation rates for desirable drug delivery regimens by modifying ONOO^—^concentration. For drug release capacity testing, PEO-b-PMATFK was loaded with vasodilator hydrochlorothiazide, which then revealed great drug delivery efficiency under ONOO^—^stimulus and near no release under other radical stimuli. With advances made in the field of sensitive and selective biomimetic nanomaterials for RONS-regulation, more attention has been paid to direct reduction of radicals implicated to cause pathology. As for RNS, newly devised polymers are often designed to directly scavenge and remove nitrogenous radicals in diseases characterized by their overproduction, particularly rheumatoid arthritis (RA). Similarly to ROS-scavenging materials, these RNS-reducing composites are often in the form of hydrogels of homologous nature (Plater et al., 2001). In 2019, Yeo et al. developed a NO-scavenging nanogel (NO-Scv gel) for alternative treatment of RA (Yeo et al., 2019). Aforementioned o-phenylenediamine group was incorporated, forming a NO-cleavable cross-linker (NOCCL) which was polymerized with acrylamide into a hydrogel. Scavenging ability is realized upon NOCCL cleavage and consumption of NO when exposed to this molecule. *In vitro* testing employing concentration range of 0–2 mg/ml evinced NO-Scv gel as having good biocompatibility and NO-scavenging capacity with low cytotoxicity. *In vivo* testing in collagen-induced arthritis (CIA) mouse model mice was performed with intra-articular injections of researched materials after immunization. 25 mg/ml of NO-Scv gel injections presented with better therapeutic outcome than 0.32 mg/ml of dexamethasone (DEXA), more efficiently quenching of NO levels in tissue and resulting in better RA onset suppression. NOCCL functionality was further employed by Kim et al. who synthesized a more sophisticated NO-scavenging and sequential drug-releasing (M-NO) gel system for the combinatorial treatment of RA (Wilson et al., 2010). Providing for rapid gelation *in situ via* a “click” cycloaddition reaction, dialkyne-functionalized-NOCCL (DA-NOCCL, *N*,*N*-(2-amino-1,4-phenylene)dipentyn-4-amide) linker has been synthesized. Next, DA-NOCCL was incorporated into an azide-functionalized hyaluronic acid backbone (HA-N_3_) and then cross-linked with azide-functionalized PEG–PLA block copolymer (PLA-*b*-PEG-N_3_). Polymerization with these units endows biocompatibility, lubricating self-healing character and drug loading capacity. Moreover, M-NO gel presented with on-demand dual-stage drug-releasing pattern, allowing for combined release of hydrophilic and hydrophobic together, further tunable by NO concentration. *In vitro* evaluation confirmed M-NO gel as being low cytotoxic and efficient NO-scavenger with resultant decrease in pro-inflammatory cytokine levels in LPS-stimulated macrophages using 50 μl of gel. *In vivo* investigation done on immunized CIA mice that received intra-articular 20 µl sample injections revealed that M-NO gel led to no significant inflammation, damage nor toxicity. Finally, as compared to other control groups, M-NO gel loaded with DEXA exhibited greatest slowing of disease progression most efficiently alleviated the arthritis symptoms in animal models (Kim et al., 2021). ROS/RNS responsive materials for implant are often biointerfaces composed of polymer antioxidants eliminate excessive ROS at the interface between living tissues and materials, and do not disturb regulated redox balance inside cells, thus eliminating unexpected cell responses, such as inflammation and dysfunction (Ikeda and Nagasaki, 2018). ROS/RNS play a fundamental role in response after the implant introduction and can influence its success. A new smart biomaterials and molecular medicine for the oxidative stress modulation in a programmable way, by the use of ROS responsive materials or by the targeting of selective molecular pathways involved in ROS generation was presented (Cerqueni et al., 2021). Moreover, neutrophil interaction with rough-hydrophilic surfaces seems to produce less proinflammatory cytokines and ROS when compared to naive smooth and rough titanium surfaces. Neutrophil responses were assessed based on adhesion, cell number, surface coverage, cell structure, cytokine secretion, ROS production, neutrophil activation, receptor expression, and neutrophil extracellular traps (NETs) release (Elangovan et al., 2022). Highly reactive free radicals, mainly ROS and RNS, damage the cell membrane, proteins, and DNA, which triggers a self-propagating inflammatory cascade of degenerative events. Dysfunctional mitochondria under oxidative stress conditions are considered a key mediator in progressive neurodegeneration. Exogenous delivery of antioxidants holds promise to alleviate oxidative stress to regain the redox balance. In this regard, natural and synthetic antioxidants have been evaluated (Ashok et al., 2022). RONS sensitive nanoparticles as a delivery system accomplish the site-specific action at the therapeutically optimal level of drug. A recent example is the development of metal chelators have also been combined with antioxidants in some advanced polymeric RONS sensitive biomaterials (Ashok et al., 2022). Ceramic and bioglasses are the main RONS sensitive biomaterials exploiting metallic elements to lower oxidative stress levels. Zinc for instance, may protect cells from oxidative protein and DNA damage as well as lipid peroxidation and improved the oxidative stress balance (Ashok et al., 2022). The appropriate delivery of the therapeutic agents, in relevant concentrations that respond to oxidative stress variations, will therefore be one the main challenges for the future development of these strategies. L-3,4-dihydroxyphenylalanine (l-dopa) was evaluated using a ROS sensitive dye, 2′,7′-diclorodihydrofluorescin (DCFH) by Luna-Velasco and coworkers (Gulcin and Alwasel, 2022). The same group used CeO_2_, Fe_2_O_3_ and Fe(0) nanoparticles to enhance ROS production during the autoxidation of L-dopa by more than four-fold in reactions that were dependent on O_2_ [71. RONS sensitive nanoparticles they could indirectly initiate the production of ROS that damage microbial cell components and viruses. Some like silver nanoparticles have broad spectrum antibacterial activity while others like cadmium containing QDs shows both antibacterial as well as antiprotozoal activity (Luna-Velasco et al., 2011). The potential toxicity of inorganic nanoparticles due to their low biodegradability, background signals interference and treatment side effects limit their clinical application. Therefore, developing biodegradable and intelligent nanoparticles are beneficial to avoid excessive metal ions deposition, specific tumor imaging and treatment (Reshma et al., 2017). Controlled RONS production was demonstrated in human mesenchymal stem cells through 1) production of nanoparticles functionalized with varying percentages of Zn(II) porphyrin and 2) modulating the number of doses of excitation light to internalized nanoparticles (Yang et al., 2020).

In this review notable developments in the field of RONS-responsive biomaterials from the past decade have been outlined, with focus on Drug Delivery Systems and radical-scavenging hydrogels. Special attention was paid to providing information on main chemical functionalities in each group of biomaterials and relating it to prominent advancements and discoveries in the field. Rapidly evolving discipline of nanomedicine with growing knowledge about reactive species, accompanied by technological progress with development of novel methods and approaches to researching and designing these molecules, creates an promising outlook for the future of RONS and numerous other materials yet to be devised. Particular potential lies in ROS- and RNS-polymers, owning to the action selectivity and sensitivity of many of those nanostructures. Supplied by wide range and variability in number of different RONS-sensitive linkers for varying molecules, it may seem like the possibilities for harnessing RONS potential for biomedical applications is infinite. However, certain challenges and limitations in the science of RONS nanochemistry can be delineated. Foremost obstacle is still insufficient knowledge about reactive species. Specifically, their exact molecular levels and interactions in different cells and tissue microenviroments remain unclear. Especially in pathological states such reliable determinations are difficult, owing to internal body milleu being a high dynamic system. Another significant issue seems result from the nature of RONS themselves. Being potent electrophiles with short half-life and high reactivity, reactive species are short-lived, minute molecules. It is why sensible generation and manipulation of RONS and their derivatives require proper storage and control of utilized materials. Finally, most pending direct problem in development of quality RONS-biomaterials is the design rationale. Virtually all probes and polymers should be specific and selective, which calls for generation of controlled, standardized and systematized materials. In addition, careful determination of biocompatibility, bioavailability, biodistribution, pharmacodynamics and pharmacokinetics, as well as toxicity. Presented challenges are significant and have to be addressed as part of individual work of researchers, as well as joint efforts of scientific communities contributing to the field. Despite abovementioned hurdles, work invested into developing RONS-regulating materials for biomedical applications will most likely continue to expand and gain in importance.

## Conclusion

Under normal physiological concentrations, reactive oxygen/nitrogen species (RONS) play a significant role in some physiological processes such as signal transduction. However, disturbances in redox balance lead to induction of oxidative stress, which in turn is associated with development of various diseases and injuries. Knowing the link between oxidative species and pathologies prompted research in the direction of developing RONS-responsive biomaterials for biomedical applications. Amongst many, some of these materials include ROS-sensitive polymers for delivery of drugs (Drug Delivery Systems, DDSs), as well as ROS-scavenging hydrogels for promotion of tissue regeneration.
